# A Neurodegenerative Vascular Burden Index and the Impact on Cognition

**DOI:** 10.3389/fnagi.2014.00161

**Published:** 2014-07-09

**Authors:** Sebastian Heinzel, Inga Liepelt-Scarfone, Benjamin Roeben, Isabella Nasi-Kordhishti, Ulrike Suenkel, Isabel Wurster, Kathrin Brockmann, Andreas Fritsche, Raphael Niebler, Florian G. Metzger, Gerhard W. Eschweiler, Andreas J. Fallgatter, Walter Maetzler, Daniela Berg

**Affiliations:** ^1^Department of Neurodegeneration, Hertie Institute for Clinical Brain Research (HIH), University of Tübingen, Tübingen, Germany; ^2^German Center for Neurodegenerative Diseases (DZNE), Tübingen, Germany; ^3^Department of Internal Medicine IV, University of Tübingen, Tübingen, Germany; ^4^Department of Psychiatry and Psychotherapy, University of Tübingen, Tübingen, Germany; ^5^Geriatric Center, University Hospital Tübingen, Tübingen, Germany

**Keywords:** vascular burden, carotid intima-media thickness, trail-making test, CERAD neuropsychological battery, prodromal marker, neurodegeneration, Alzheimer’s disease, Tübinger evaluation of risk factors for early detection of neurodegeneration (TREND) study

## Abstract

A wide range of vascular burden factors has been identified to impact vascular function and structure as indicated by carotid intima–media thickness (IMT). On the basis of their impact on IMT, vascular factors may be selected and clustered in a vascular burden index (VBI). Since many vascular factors increase the risk of Alzheimer’s disease (AD), a multifactorial neurodegenerative VBI may be related to early pathological processes in AD and cognitive decline in its preclinical stages. We investigated an elderly cohort at risk for neurodegeneration (TREND study, *n* = 1102) for the multifactorial influence of vascular burden factors on IMT measured by ultrasound. To create a VBI for this cohort, vascular factors and their definitions (considering medical history, medication, and/or blood marker data) were selected based on their statistical effects on IMT in multiple regressions including age and sex. The impact of the VBI on cognitive performance was assessed using the Trail-Making Test (TMT) and the consortium to establish a registry for Alzheimer’s disease (CERAD) neuropsychological battery. IMT was significantly predicted by age (standardized β = 0.26), sex (0.09; males > females) and the factors included in the VBI: obesity (0.18), hypertension (0.14), smoking (0.08), diabetes (0.07), and atherosclerosis (0.05), whereas other cardiovascular diseases or hypercholesterolemia were not significant. Individuals with 2 or more VBI factors compared to individuals without had an odds ratio of 3.17 regarding overly increased IMT ( ≥ 1.0 mm). The VBI showed an impact on executive control [log(TMT B−A), *p* = 0.047] and a trend toward decreased global cognitive function (CERAD total score, *p* = 0.057) independent of age, sex, and education. A VBI established on the basis of IMT may help to identify individuals with overly increased vascular burden linked to decreased cognitive function indicating neurodegenerative processes. The longitudinal study of this risk cohort will reveal the value of the VBI as prodromal marker for cognitive decline and AD.

## Introduction

The circulatory and metabolic functions of the vascular system underlying the reliable supply of oxygen and nutrients can be compromised by a wide range of factors. Pathological processes as well as lifestyle factors may affect vascular structure and function during life span. For instance, cardiovascular diseases, atherosclerosis, stroke, diabetes, hypertension, as well as smoking, obesity, hypercholesterolemia, and lack of physical activity have been associated with altered vascular structure (Salonen and Salonen, 1991; Folsom et al., 1994; Bots et al., 1997; van Popele et al., 2001; Urbina et al., 2002; Kotsis et al., 2006; Baldassarre et al., 2010; Engelen et al., 2012; Niu et al., 2013; van den Oord et al., 2013). Specifically, these studies showed an association of these vascular factors with structural increases in the intima–media thickness (IMT) of the common carotid artery (CCA). Increased IMT has been shown to serve as an indicator of vascular age (Stein et al., 2004) and arterial stiffness (van Popele et al., 2001), and to represent a strong predictor of clinical cardiovascular events (Lorenz et al., 2007). Clustering of multiple factors into a cumulative vascular burden index (VBI) has been shown to predict IMT better than single factors do (Urbina et al., 2002; Baldassarre et al., 2010; Niu et al., 2013) suggesting a converging impact of multiple different factors on IMT.

Other indices have been established comprising (cardio)vascular as well as other factors, such as age and sex, to estimate the annual risk of stroke for patients with atrial fibrillation (CHA_2_DS_2_-VASc score; Olesen et al., 2011) or the 10-year cardiovascular risk of an individual (Framingham risk score; D’Agostino et al., 2008). While these (cardio)vascular risk indices have a high validity and specificity for particular diseases, they may not indicate vascular burden as relevant for other pathologies, e.g., neurodegenerative disease such as Alzheimer’s disease (AD).

For the risk evaluation of AD an individual quantification of vascular burden mediated through multiple vascular factors associated with AD is crucial for two main reasons. (1) Chronic brain hypoperfusion leading to cerebral hypoxia represents an important aspect of neurodegenerative processes in AD and vascular dementia (Breteler, 2000; Zlokovic, 2005; de la Torre, 2012b), and often precedes the first cognitive symptoms by many years (Chao et al., 2010; de la Torre, 2012a). (2) Many vascular factors such as hypertension, diabetes, obesity, hypercholesterolemia, smoking, or stroke have been shown to increase the AD risk (Breteler, 2000; Honig et al., 2003; Kivipelto et al., 2005; Kivipelto and Solomon, 2006; Purnell et al., 2009; Cataldo et al., 2010) and the conversion from mild cognitive impairment (MCI) to AD (Li et al., 2011). A VBI may therefore be of particular importance for longitudinal studies, where vascular burden may predict AD or help to detect its early preclinical stages.

Decreased cognitive function as assessed using well-established neuropsychological tests is characteristic for MCI and AD and key to their diagnosis (Hort et al., 2010). Neurodegenerative processes as well as cognitive decline precede the clinical diagnosis of MCI and AD (Smith et al., 2012; Langbaum et al., 2013; Moghekar et al., 2013; Lim et al., 2014). Given the important role of vascular burden factors in the etiology of AD, the impact of a VBI on cognitive function in non-demented elderly individuals may validate its relevance for the early detection of MCI and AD.

In the present study, we used vascular risk factors of AD showing an impact on IMT to establish a VBI in a large cohort of non-demented, elderly individuals [Tübinger evaluation of risk factors for early detection of neurodegeneration (TREND) study, *n* = 1102, 51–84 years, (Hobert et al., 2011; Berg, 2012; Maetzler et al., 2012; Heinzel et al., 2013; Gaenslen et al., 2014)]. We hypothesized that this multifactorial VBI may contribute to neurodegenerative aging processes. To validate this link, we investigated this cohort at risk for neurodegeneration for the impact of the VBI on cognitive performance in neuropsychological tests.

## Materials and Methods

### Participants

All individuals included in the present analyses (*n* = 1102; 528 female, 574 male) participated in the first follow up of the TREND study (March 2011 to April 2012), which represents the baseline assessment with regard to the IMT ultrasound measurements. The prospective and longitudinal TREND study aims to identify risk markers for the prediction of AD and Parkinson’s disease (PD), respectively, in non-demented elderly individuals who are investigated every 2 years. Participants were partly selected due to the presence of prodromal risk markers of AD and PD, respectively, including REM-sleep disorder, hyposmia, and/or depression. For a detailed outline of the TREND study, further inclusion and exclusion criteria and TREND baseline assessments, see Berg (2012). The study was approved by the ethical committee of the Medical Faculty of the University of Tübingen (Nr. 90/2009BO2). All procedures were in accordance with the Declaration of Helsinki in its latest version, and all participants gave written informed consent.

### Vascular burden index

In order to establish a VBI for the TREND cohort, we first investigated AD-associated vascular risk factors with different possible definitions (see below) for their effect size regarding IMT prediction. We then selected single factors and definitions yielding the best prediction of IMT (independent of age and sex) in multiple regression analyses (see Statistics). The cumulative number of these selected factors/definitions was used as VBI.

### Vascular burden factors

Vascular risk factors, diseases and medication data of the TREND study were assessed with questionnaires and personal medical history interviews. The following factor definitions were included in the analyses (see also Table 1).

**Table 1:** **Multiple regression analyses of intima-media thickness (IMT) and single putative vascular burden factors**.

Vascular factor	Alternative factor definitions	Definition-dependent subgroup size (indicated/total)	Predictor statistics					Full regression model	
			β	B	SE(B)	*t*-Value	*p*-Value	*R*^2^	*F*-value
Diabetes	Medical history	83/1102	0.067	0.039	0.017	2.30	0.021	0.088	35.30
	Medical history or medication	86/1102	0.065	0.038	0.017	2.25	0.025	0.088	35.22
	Medical history or medication or HbA1c^a^	102/1102	0.074	0.040	0.016	2.56	0.011	0.089	35.75
Hypertension	Medical history^a^	454/1102	0.138	0.043	0.009	4.73	<0.001	0.102	41.52
	Medication	463/1102	0.102	0.032	0.009	3.44	0.001	0.093	37.67
	Medical history and medication	408/1102	0.126	0.040	0.009	4.28	<0.001	0.099	40.05
	Medical history or normal with medication	593/1102	0.115	0.036	0.009	3.93	<0.001	0.096	39.00
Hypercholesterolemia	Medical history	460/1102	0.028	0.009	0.009	0.96	0.337	0.084	33.71
	Medication	201/1102	0.013	0.005	0.012	0.46	0.647	0.084	33.45
	Medical history and medication	156/1102	0.017	0.008	0.013	0.59	0.556	0.084	33.50
	Medical history or normal with medication	505/1102	0.026	0.008	0.009	0.89	0.371	0.084	33.67
Cardiovascular disease/cardiac conditions	Atherosclerosis^a^	57/1102	0.051	0.036	0.020	1.75	0.080	0.086	34.49
	Atrial fibrillation	44/1102	−0.027"	−0.022"	0.023	−0.93”	0.353	0.084	33.69
	Cardiac arrhythmia	121/1102	−0.012"	−0.006"	0.014	−0.41”	0.682	0.084	33.44
	Myocardial infarction	34/1102	−0.033"	−0.030"	0.026	−1.14	0.255	0.085	33.85
	Congestive heart failure	35/1102	−0.001"	−0.001"	0.026	−0.003”	0.973	0.084	33.38
	Coronary heart disease	48/1102	0.003	0.003	0.022	0.12	0.906	0.084	33.38
Obesity	BMI >30^a^	156/1092	0.176	0.078	0.013	6.15	<0.001	0.116	47.47
	BMI >35	32/1092	0.104	0.096	0.027	3.59	<0.001	0.097	39.09
Smoking	Pack years >5	282/1062	0.034	0.012	0.011	1.13	0.259	0.084	32.15
	Pack years >10	228/1062	0.070	0.027	0.011	2.37	0.018	0.087	33.72
	Pack years >15^a^	156/1062	0.076	0.034	0.013	2.57	0.010	0.088	34.09
	Pack years >20	115/1062	0.072	0.037	0.015	2.44	0.088	0.088	33.86

*^a^Indicates the factor definition with the largest β-value and thus selected VBI factor; β indicates the standardized regression coefficient of the predictor in the multiple regression model; B indicates the non-standardized β; and SE(B) its standard error*.

#### Diabetes

Life-time diagnosis of diabetes (factor label: medical history), additional intake of antidiabetic medication (medical history or medication), and glycated hemoglobin (HbA_1c_) levels of HbA_1c_ ≥ 6.5% (Kilpatrick et al., 2009) (medical history or medication or HbA_1c_) were considered as factors definitions.

#### Hypertension

Life-time diagnosis of hypertension (medical history), intake of anti-hypertensive medication alone (medication), diagnosis and medication intake (medical history and medication), and diagnosis and non-diagnosis with medication intake (medical history or normal with medication) alternatively defined the factor hypertension.

#### Hypercholesterolemia

Positive medical history and/or intake of lipid-lowering medication were considered for factor definitions.

#### Cardiovascular disease/cardiac conditions

Life-time diagnosis of atherosclerosis, atrial fibrillation, cardiac arrhythmia, myocardial infarction, congestive heart failure, and coronary heart disease, were each independent factor definitions.

#### Obesity

A body mass index (BMI; mass [kg]/(height [m])^2^) above 30 (obese class I) and BMI >35 (obese class II) was considered. BMI data of 10 participants were missing.

#### Smoking

Personal history of smoking behavior was indicated by “pack years” quantifying the packs (of 20 cigarettes) smoked per day multiplied by years as a smoker. Due to lack of consensus, we arbitrarily set the factor threshold to 5, 10, 15, and 20 pack years as factor definitions. For 40 former smokers, pack years data were not available.

Due to lack of vascular factor data the VBI could not be calculated for a total of 50 individuals.

### Ultrasonography

We performed ultrasonographic measurements of the right CCA using an ACUSON Antares™ ultrasound system (Siemens, Erlangen, Germany) and a 5–10 MHz linear array transducer (VF10-5, Siemens, Erlangen, Germany). Participants were examined in a supine position. The CCA was differentiated from the internal jugular vein in a transverse plane and then displayed in a longitudinal scan while the course of the CCA was followed to the carotid bulb. The IMT of the CCA was defined as the distance between the echogenic intima-line and the echogenic media–adventitia border at the far-wall of the CCA (Touboul et al., 2012). IMT was measured 1 cm proximal to the carotid bulb in a segment free of plaque using a fourfold magnification of the ultrasound image (Engelen et al., 2012). After visual inspection image quality at the site of measurement was verified by an experienced sonographer.

### Cognitive testing

Participants performed established and standardized cognitive tests including the Trail-Making Test (TMT; Corrigan and Hinkeldey, 1987; Bowie and Harvey, 2006; Sanchez-Cubillo et al., 2009) and the consortium to establish a registry for Alzheimer’s disease (CERAD) neuropsychological battery (Morris et al., 1989).

The administered TMT is a paper-and-pencil task and consists of two parts: for the TMT Part A participants connect randomly distributed numbers (1–25) in ascending numerical order. For the TMT Part B participants connect randomly distributed numbers (from 1 to 13) and letters (from A to L) in alternating numeric and alphabetical order (1–A–2–B–3–C– … –13–L). In case of errors, immediate feedback is provided by the examiner, so that the participant completes the task without errors (at the expense of additional time) (Bowie and Harvey, 2006). TMT performance was indicated by the time [s] for completing TMT Part B minus the time needed for TMT Part A. The difference TMT B−A represents an effective performance measure of cognitive control and executive functioning, where potential bias due to differences in upper extremity motor speed, simple sequencing, visual scanning, and psychomotor functioning are minimized (Corrigan and Hinkeldey, 1987; Sanchez-Cubillo et al., 2009).

The CERAD is composed of five subtests including animal naming, modified Boston Naming Test, Mini-Mental State Examination (MMSE), constructional praxis, and word list memory, which have been shown to represent valid and reliable measures of cognitive performance in the elderly and in AD patients (Welsh-Bohmer and Mohs, 1997). The total score of the CERAD battery has been shown to provide an effective global measure of cognitive functioning for the English as well as for the German version of the CERAD used in the present study (Chandler et al., 2005; Ehrensperger et al., 2010). The total score ranges between 0 and 100 and comprises the raw scores of the CERAD animal naming (verbal fluency; maximum of 24 points), modified Boston Naming Test (15 points), word list learning (30 points), constructional praxis (11 points), word list recall (10 points), and word list recognition discriminability (10 points) (Chandler et al., 2005; Ehrensperger et al., 2010).

### Statistics

First, we aimed to identify vascular burden factors (with the definition yielding the largest effect size), which predict IMT independent of age and sex. Therefore, we calculated multiple regressions (inclusion algorithm) with IMT as criterion variable, and age, sex (dummy variable: female = 0, male = 1), and the vascular burden factor of interest as predictors. Vascular burden factors showing a statistical trend (*p* < 0.1) of prediction of increased IMT were included in the VBI. For these factors the definition/threshold of the highest effect size as measured by the standardized β coefficient of the predictor was chosen.

Second, we investigated the association of the VBI with (pathological) IMT extremes. Current guidelines suggest an IMT of 0.9 mm as a conservative estimate of common carotid IMT abnormalities (Mancia et al., 2007). Therefore, we calculated the odds ratio (OR) and the 95% confidence interval (CI) comparing individuals without vascular burden factors with individuals possessing 1 or 2+ factors, respectively, and their frequency of falling into the 75% (IMT <0.9 mm) and 90% percentile (IMT <1.0 mm) of the IMT distribution of the sample.

Third, we analyzed the impact of the VBI on cognitive performance. Univariate analyses of covariance using the TMT (log_10_[TMT B−A]; logarithmized due to non-normality) or the CERAD total score as dependent variables, the VBI (0, 1, 2+ vascular burden factors) as between-subject factor, and age, sex, and education as covariates were performed. Education level was coded according to the International Standard Classification of Education (ISCED-97) of the UNESCO (http://www.unesco.org/) as low (level 1–2; primary education and secondary education first stage), medium (3–4; secondary and post-secondary, non-tertiary education), and high (5–6; tertiary education).

*Post hoc t*-tests were performed and the significance threshold was set to α <5%, and to α <10% for statistical trends for all tests.

## Results

### Sample characteristics

The mean age (±SD) of the cohort was 65.1 ± 6.8 years (range: 50.8–83.7 years). Compared to males (66.0 ± 6.7; 51.6–82.2), females (64.4 ± 6.8; range: 50.8–83.7) were younger (*t*_1100_ = −4.09, *p* < 0.001). Males had higher education levels (low: 0.9%, medium: 49.5%, and high: 49.5%) than females (11.2%, 61.4%, and 27.5%; χ^2^ = 91.55, *p* < 0.001).

### Intima–media thickness

The IMT had a mean value of 0.76 ± 0.16 mm. Females had lower IMT values than males (0.74 ± 0.15 mm versus 0.77 ± 0.16 mm, *t*_1100_ = −4.00, *p* < 0.001).

### Effects of single vascular factors on IMT

The prevalence of the vascular burden factors is given in Table 1. IMT was significantly predicted by age (mean β-value (±SD) of the 23 multiple regressions: 0.262 ± 0.011, *p* < 0.001) as well as sex (0.087 ± 0.006, *p* < 0.05).

Independent of these age and sex effects several vascular burden factors significantly predicted IMT. Obesity (BMI >30) was the strongest IMT predictor (β = 0.176), followed by hypertension (medical history; β = 0.138), smoking (pack years >15; β = 0.076), and diabetes (medical history/medication + HbA_1c_; β = 0.074), while atherosclerosis showed a statistical trend of IMT prediction (β = 0.051, *p* = 0.080). These vascular burden factors were therefore selected to be included in the VBI. The full regression models [including the predictors age, sex, and single (significant) vascular burden factors] explained between 8.6 and 11.6% of the IMT variance.

No significant IMT prediction was found for other cardiovascular diseases/conditions (*p* > 0.3) or hypercholesterolemia (*p* > 0.3) in regression models including age and sex as predictors.

### Vascular burden index in association with IMT

**Figure 1:**
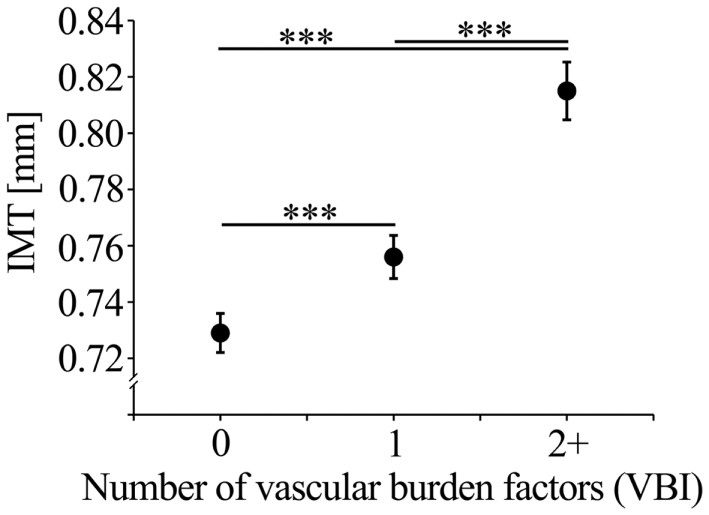
**Mean IMT (with standard error of the mean; SEM) corrected for age, sex, and education of the VBI groups**.

The number of selected vascular burden factors (VBI) showed an effect size of β = 0.203 with regard to IMT prediction independent of age and sex. The full model (age, sex, VBI) explained 12.4% of IMT variance. For the VBI groups (0, 1, 2+ vascular burden factors), the mean IMT values corrected for age and sex are shown in Figure 1. The VBI was correlated with age (Spearman ρ = 0.11, *p* < 0.001), but did not differ between males and females (logistic regression including age; *p* = 0.53).

After selection of the single vascular burden factors on the basis of their effects on IMT the impact of the cumulative number of these factors (VBI) on pathological IMT values was investigated. The VBI was significantly related to the frequency of individuals being in the upper 25% ( ≥ 0.9 mm; χ^2^ = 35.77, *p* < 0.001) or the upper 10% ( ≥ 1.0 mm; χ^2^ = 27.62, *p* < 0.001) of the IMT distribution. Individuals with one compared with those without vascular burden factors had an OR of 1.68 (95% CI: 1.21–2.33) of being in the upper 25% of the IMT distribution. However, for individuals with two or more compared to those without vascular burden factors an increased OR for the upper 25% of 2.97 (95% CI: 2.06–4.28), and 3.17 (95% CI: 1.95–5.16) for the upper 10% of the IMT distribution was observed.

### Vascular burden index in association with cognitive performance

**Figure 2:**
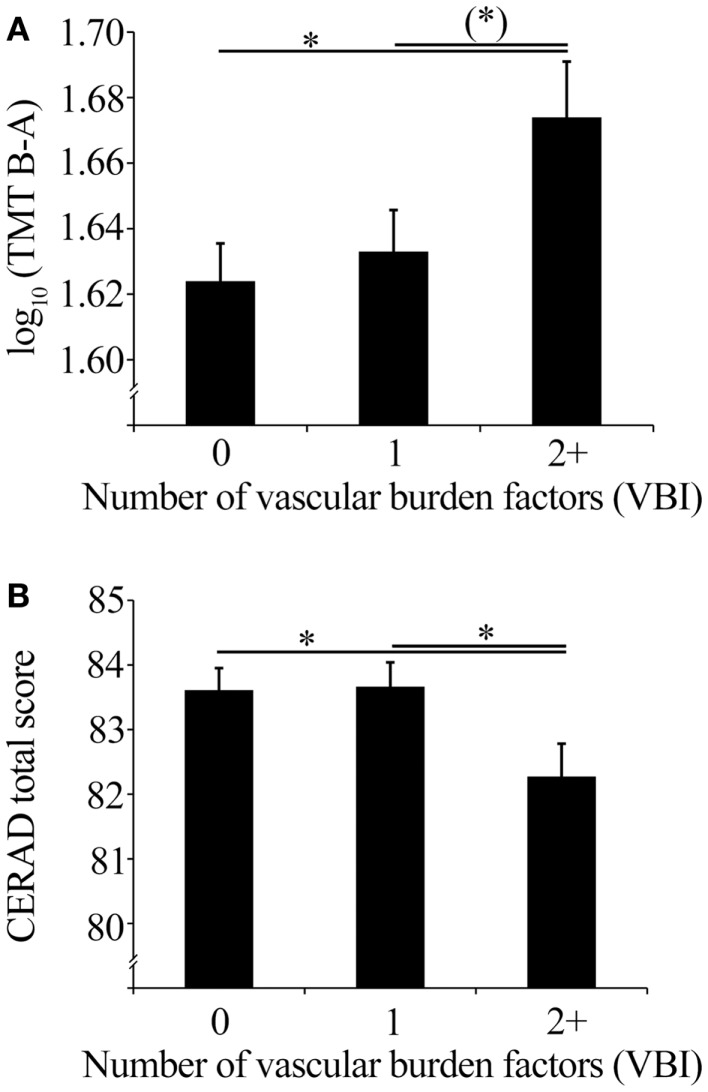
**(A)** Mean TMT performance (with SEM) corrected for age, sex, and education of the VBI groups, and **(B)** mean CERAD total scores (with SEM) corrected for age, sex, and education of the VBI groups.

The VBI significantly impacted cognitive control and executive function as indicated by TMT performance (*F*_2, 1033_ = 3.08, *p* = 0.047). *Post hoc* tests showed that individuals with two or more vascular burden factors showed lower TMT performance (i.e., longer time for TMT B−A) compared to individuals without these factors (*p* = 0.015). Compared to those individuals with one vascular burden factor this performance difference showed a statistical trend (*p* = 0.051). Mean values of TMT performance corrected for age, sex, and education are shown in Figure 2A.

Global cognitive functioning as indicated by the CERAD total score showed a statistical trend of an impact of the VBI (*F*_2, 1043_ = 2.88, *p* = 0.057). Individuals with two or more vascular burden factors showed decreased global cognitive performance compared to individuals without (*p* = 0.030), or with one vascular burden factor (*p* = 0.028). CERAD total score mean values corrected for age, sex, and education are shown in Figure 2B.

Unlike the VBI, the presence of IMT pathology (i.e., IMT ≥0.9 and IMT ≥1.0, respectively) showed no significant impact on cognitive performance measures (*p* > 0.10) independent of age, sex, and education.

## Discussion

In the present study, a VBI based on vascular risk factors previously associated with AD was established for a large cohort of 1102 elderly individuals at risk for neurodegenerative diseases (TREND study). Based on the impact of single (cardio)vascular risk factors on IMT vascular burden factors were selected to be included in a cumulative VBI. Moreover, the relevance of this VBI for IMT extremes as well as for cognition in the TREND cohort was investigated.

### Single vascular burden factors

The cumulative VBI established in this study comprises the factors obesity (standardized β = 0.176), hypertension (β = 0.138), diabetes (β = 0.074), smoking (β = 0.076), and atherosclerosis (β = 0.051), which predicted IMT independent of age (β = 0.262) and sex (β = 0.087). However, effect sizes were small and together with age and sex the single factors only explained between 8.6 and 11.6% of the IMT variance.

Previous studies investigating individuals in a similar age range have consistently reported an impact of these single vascular factors on IMT. For age comparable effect sizes have been shown (β = 0.276) (Rundek et al., 2013). Also, males have been shown to have slightly higher IMT values than females (β = 0.150) (Rundek et al., 2013).

For the BMI, a similar effect size of IMT prediction (β ~ 0.1) has been reported for elderly adults (Engelen et al., 2012). An IMT difference of about 0.1 mm between adult normal-weighted and obese individuals has been previously reported (Kotsis et al., 2006), which corresponds to the effects of class I obesity (BMI >30) in the present sample.

The small effect size of life-time diagnosis of hypertension in the present sample is comparable to the previously reported small effect size of IMT prediction by systolic blood pressure (β ~ 0.1) (Engelen et al., 2012), or IMT differences between elderly normotensive and hypertensive individuals (Bots et al., 1993).

For diabetes predicting increased IMT similar effect sizes in elderly individuals with cardiovascular diseases (β ~ 0.10) and a slightly higher effect size (β ~ 0.19) in a healthy elderly population has been reported (Engelen et al., 2012).

Smoking as defined by more than 15 pack years showed a slightly smaller effect size as previously reported for pack years smoking (β = 0.125) (Salonen and Salonen, 1991). However, for current smoking effect sizes were shown to differ between healthy, elderly individuals and sexes (men: β = 0.25, women: β = 0.11) and elderly individuals with cardiovascular diseases (men: β = 0.10, women: β = −0.10) (Engelen et al., 2012). Thus, the impact of the factor smoking on IMT might be complex and sex-dependent.

Contrary to numerous previous studies associating atherosclerosis with increased IMT (Bots et al., 1996, 1997; van Popele et al., 2001), we only found a small effect of the factor atherosclerosis (β = 0.051) on IMT. However, effect sizes of reported life-time atherosclerosis (*n* = 57) may be small as effects on IMT prediction were calculated to be independent of age and sex.

Other cardiovascular diseases and conditions were no significant IMT predictors in the present study, which is in contrast to several previous findings showing increased IMT for e.g., myocardial infarction, congestive heart failure, coronary heart disease (Bots et al., 1997; Den Ruijter et al., 2012; van den Oord et al., 2013). The missing IMT effects in the present study might be due to the younger age and rather small number of individuals with these cardiovascular pathologies, and age and sex being confounding factors in multiple regression analyses.

Life-time diagnosis of hypercholesterolemia was no significant IMT predictor independent of age and sex in the present study. Recently, very small effects (β < 0.05) of IMT prediction have been reported for the Total-to-HDL cholesterol ratio in elderly individuals (Engelen et al., 2012). Also, life-time hypercholesterolemia effects on IMT might have been ameliorated due to lipid-lowering treatment or nutritional changes in the past.

### Vascular burden index

The number of vascular burden factors (VBI) together with age and sex explained 12.4% of the IMT variance. This percentage corresponds to previous large studies in elderly individuals, which showed 11% (Rundek et al., 2013), 17% (O’Leary et al., 1996), or 27% (Baldassarre et al., 2010) of IMT variance to be explained by age, sex, and vascular burden factors (e.g., blood pressure, pack years or current smoking, serum cholesterol or glucose levels, and others) and factors, such as education or geographical data. However, composition of these factors included in these studies did widely differ, and in the study by Baldassarre et al. (2010) the IMT predictor with the largest effect size was geographical latitude within Europe (partial *r* = 0.078), which partly explains the large percentage (27%) of explained IMT variance.

Thus, variance of IMT as a continuous variable including non-pathological IMT values ( <0.9 mm) and (heritable) natural differences between individuals might only be modestly explained by vascular burden factors. This might also underlie the modestly increased IMT prediction comparing the VBI (12.4%) and the single vascular factor of largest effect size (obesity: 11.6%). However, the impact of vascular burden factors might be better observed for pathological IMT extremes. Here, we found a pronounced impact of the number of vascular burden factors with respect to the extreme subgroups. For instance, individuals with two or more compared to those without vascular burden factors showed an increased OR of 2.97 for IMT ≥0.9 mm, and an OR of 3.17 for IMT ≥1.0 mm. These findings suggest that within normal, non-pathological IMT ranges (i.e., IMT <0.9 mm) natural and largely age- and sex-dependent interindividual IMT variability might prevail, whereas the impact of the VBI is best observed in subgroups of increased IMT.

The VBI was established for the TREND cohort to investigate its predictive value for MCI and AD in future longitudinal investigations of the TREND study. Importantly, in elderly individuals, in whom cerebral perfusion is already diminished due to aging a high vascular load, e.g., mediated by two, three, or more vascular burden factors, might causally underlie chronic brain hypoperfusion, hypoxia, and a neuronal energy crisis critical with respect to cellular functioning and may, hence, foster neurodegeneration increasing the risk of AD (Li et al., 2011; de la Torre, 2012a). Because chronic brain hypoperfusion often precedes MCI and AD by many years, the present study identified individuals hypothesized to show cognitive decline in the future due to vascular burden factors. A recent study even showed a high association of increased pulse wave velocity with beta-amyloid burden in elderlies (Hughes et al., 2013) suggesting an important link between vascular and beta-amyloid mediated pathological processes relevant to AD.

### Vascular burden index and cognition

The present study showed an impact of the VBI on cognition. Individuals with two or more vascular burden factors exhibited poorer executive function/cognitive control in the TMT and lower global cognitive function in the CERAD neuropsychological battery compared to individuals without these factors, which was independent of age, sex, and education. This finding indicates that the VBI is indeed associated with cognitive performance in this study cohort. As associations between cognitive performance and IMT were not significant in the present study, the VBI may represent a more reliable and holistic indicator of vascular impact on cognition, compared to (pathological) IMT alone.

Importantly, the VBI comprises vascular factors which have previously been associated with AD. Also, by considering objective and quantitative IMT measurements for the selection of these factors and factor definitions, the validity of the VBI indicating vascular burden as relevant for AD in individuals of the TREND cohort might be increased. Thus, the VBI may hold important predictive value for cognitive decline and the development of MCI and AD, which will be further investigated in the longitudinal TREND study.

### Limitations

Several limitations have to be considered for the present study: (1) by selecting vascular factors for the VBI based on IMT some putative factors/definitions were not included in the VBI. This procedure may increase sensitivity of the VBI as selected factors actually predict increased IMT in the TREND cohort but may also reduce its specificity by excluding factors (e.g., cardiovascular pathologies, hypercholesterolemia). (2) We only used questionnaires and personal medical history interviews for the assessment of vascular risk factors, diseases, and medication data. However, it was emphasized that all indications, diagnoses, and medications must have been confirmed by a medical doctor. Moreover, experimental blood pressure measurements were not included in the establishment of the VBI and the identification of individuals with increased vascular burden. Reliable (multiple) measurements of blood pressure or other experimental vascular and laboratory measures should be investigated in future studies for their potential to further validate or refine vascular burden estimates, such as the VBI. (3) While total or HDL cholesterol, or fasting glucose levels or other blood-derived quantitative measures were not assessed due to assessments at different day times, we aimed to complement the data by also considering current medication and, for diabetes, blood HbA_1c_ concentrations. (4) We only assessed one frequently investigated segment of the IMT of the right CCA as a reference measure of vascular burden, as the design of the TREND study favors easy to apply and quick to perform techniques, which may well be used in other large studies or populations screenings. However, other indicators including internal carotid artery measures (Mackinnon et al., 2004), regional plaques (Zureik et al., 2002), and arterial stiffness (van Popele et al., 2001; Hughes et al., 2013) were not investigated, but might also be important for the relationship between vascular burden factors and vascular functionality. (5) Interactions between vascular burden factors were not in the scope of the present study. However, their interactions have been shown to only modestly improve explained IMT variance (Niu et al., 2013). (6) The present results might not be fully generalizable, as TREND study participants had above average education levels (39% academics) and were partly selectively included due to the presence of prodromal markers for neurodegeneration (REM-sleep disorder, hyposmia, and/or depression) (Berg, 2012).

## Conclusion

Vascular function and structure as well as the risk of AD have been shown to be influenced by a multitude of vascular factors. Based on their impact on carotid IMT vascular factors associated with AD can be selected to establish a neurodegenerative cumulative VBI. The relevance of this VBI for cognition was validated by its impact on cognitive performance in healthy, non-demented individuals at risk for neurodegeneration. The VBI may represent an important predictor of AD and its prodromal stages, which will be further investigated in the longitudinal TREND study.

## Author Contributions

Conceived and designed the experiments and edited the manuscript: Gerhard W. Eschweiler, Andreas J. Fallgatter, Walter Maetzler, Daniela Berg. Performed the experiments: Benjamin Roeben, Isabel Wurster, Kathrin Brockmann, Raphael Niebler, Florian G. Metzger, Andreas Fritsche. Analyzed the data: Sebastian Heinzel, Inga Liepelt-Scarfone, Benjamin Roeben, Ulrike Suenkel, Isabella Nasi-Kordhishti. Wrote the manuscript: Sebastian Heinzel, Inga Liepelt-Scarfone, Walter Maetzler, Daniela Berg. All authors were involved in interpretation of the data and critical revision of the manuscript, all authors gave their final approval.

## Conflict of Interest Statement

The authors declare that the research was conducted in the absence of any commercial or financial relationships that could be construed as a potential conflict of interest.
